# Role of UPR Pathway in Defense Response of *Aedes aegypti* against Cry11Aa Toxin from *Bacillus thuringiensis*

**DOI:** 10.3390/ijms14048467

**Published:** 2013-04-17

**Authors:** Leidy P. Bedoya-Pérez, Angeles Cancino-Rodezno, Biviana Flores-Escobar, Mario Soberón, Alejandra Bravo

**Affiliations:** Instituto de Biotecnología, Universidad Nacional Autónoma de México. AP 510-3, Cuernavaca 62250, Morelos, Mexico; E-Mails: lbedoya@ibt.unam.mx (L.P.B.-P.); angelescancino@gmail.com (A.C.-R.); biviana@ibt.unam.mx (B.F.-E.); mario@ibt.unam.mx (M.S.)

**Keywords:** Cry toxins, *Bacillus thuringiensis*, UPR, SREBP, defense responses

## Abstract

The insecticidal Cry toxins are pore-forming toxins produced by the bacteria *Bacillus thuringiensis* that disrupt insect-midgut cells. Cells can trigger different survival mechanisms to counteract the effects of sub-lytic doses of pore forming toxins. Particularly, two signaling pathways have been demonstrated to play a role in the defense mechanism to other toxins in *Caenorhabditis elegans* and in mammalian cells. These are the unfolded protein response (UPR) and the sterol regulatory element binding proteins (SREBP) pathways, which are proposed to facilitate membrane repair responses. In this work we analyzed the role of these pathways in *Aedes aegypti* response to intoxication with Cry11Aa toxin. We show that UPR is activated upon toxin ingestion. The role of these two pathways was analyzed *in vivo* by using RNA interference. We silenced the expression of specific proteins in *A. aegypti* larvae. Gene silencing of Ire-1 and Xbp-1 proteins from UPR system, resulted in hypersensitive to Cry11Aa toxin action. In contrast, silencing of Cas-1, Scap and S2P from SREBP pathway had no affect on Cry11Aa toxicity in *A. aegypti* larvae. However, the role of SREBP pathway requires further studies to be conclusive. Our data indicate that the UPR pathway is involved in the insect defense against Cry toxins.

## 1. Introduction

The three-domain Cry (3d-Cry) toxins produced by *Bacillus thuringiensis* bacteria are insect pathogenic proteins. The 3d-Cry toxins are pore-forming toxins that affect the midgut cells of their insect hosts [1]. They are specific since they interact with specific proteins located in apical membrane of insect midgut cells. The sequential interaction with these toxin receptors facilitates the oligomerization of the toxin and its insertion into the membrane, forming a pore that kills the cells and the larvae. 3d-Cry toxins are currently used as an efficient control practice of insect-pests worldwide and have helped to reduce the use of chemical insecticides [2].

The 3d-Cry toxins show toxicity to different insect species belonging to several insect orders such as Coleoptera, Lepidoptera and Diptera, which are important pests in agriculture or in public health, since they attack main agricultural crops or are vectors of important human-diseases such as dengue and malaria [1].

In this work we studied the host responses to toxin action, specifically the responses of the mosquito *Aedes aegypti* upon intoxication with Cry11Aa toxin from *B. thuringiensis* subsp. *israelensis*. Several studies have reported that cells trigger different survival mechanisms to counteract the effects of sub-lytic doses of pore forming toxins. These host responses may include adaptive or innate immunity responses as well as cellular non-immune defenses [3]. Cells are able to sense the changes in ion concentrations and repair the damage in their plasma membrane. Different responses have been documented in insects [4], nematodes [5,6] and mammalian cells [7–9]. Among these responses the activation of signaling pathways such as mitogen activated kinases p38 and JNK, caspase-1 that induces activation of the inflammasome and sterol regulatory element binding proteins (SREBP), autophagy and unfolded protein responses (UPR) have been shown to participate in overcoming toxin effects and promoting cell survival [4–10].

We have previously shown that MAPK p38 participates in the defense to Cry1Ab toxin in the Lepidoptera *Manduca sexta* and to Cry11Aa toxin in the Diptera *A. aegypti*[4]. We became interested in the study of the UPR and SREBP responses, since both have been proposed to challenge toxin action by facilitating membrane repair in *Caenorhabditis elegans* and in mammalian cells, when they were exposed to Cry5Ba toxin or to aerolysin respectively [5,7]. We determined if similar mechanisms exist in insects to protect them from the action of 3d-Cry toxins.

The UPR system responds to unfolded proteins in the lumen of the endoplasmic reticulum (ER) by activating at least three different signal transduction pathways that are mediated by ATF-6, PERK and IRE-1 [11]. It was shown that in particular the IRE-1 arm of UPR system is activated upon intoxication with pore forming toxins in the nematode and in mammalian cells and this response was directly related to survival [5].

Proteins of the SREBPs pathway are membrane bound transcription factors that initially reside in ER and form a complex with SCAP protein. The SREBP-SCAP complex is transported to Golgi apparatus where they are cleaved by specific proteases, named S1P and S2P, to release the transcription factor domain [12]. The SREBP system is activated after infection of mammalian cells with aerolysin promoting cell survival most probably by the upregulation of lipogenic genes involved in membrane repair [7,9].

In this work we used dsRNA interference system to silence expression of specific proteins of these two signaling pathways in *A. aegypti* larvae in order to analyze if they play a role *in vivo* against the action of Cry11Aa toxin.

## 2. Results

### 2.1. Role of IRE-1 UPR Pathway in Response to Cry11Aa in *A. aegypti*

**Figure 1:**
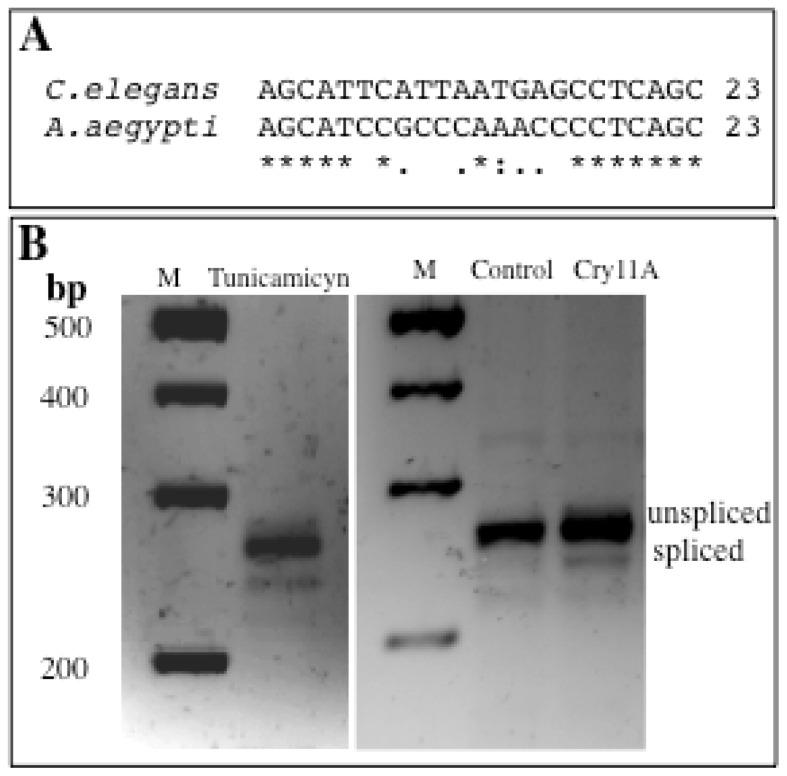
The splicing of *xbp*-1 by the IRE-1 kinase/endonuclease is induced as response to Cry11Aa intoxication in *Aedes aegypti*. (**A**) Alignment of the sequence of the 23 nucleotides that constitutes the intron of *xbp*-1 that is cleaved in *C. elegans* and the corresponding sequence in *A. aegypti*; (**B**) Splicing of *xbp*-1 is induced in *A. aegypti* by tunicamicyn or after 2 h of intoxication with Cry11Aa toxin at LC_50_. M, nucleotide size markers. Control, were larvae feed without toxin.

IRE-1 (inositol requiring enzyme 1) is part of the UPR system response to stress situations, that was directly associated with stress induced by pore forming toxins [3,5]. IRE-1 is a highly conserved protein with a dual function as kinase/endonuclease that cleaves out an intron of 23 nucleotides in the mRNA of the transcription factor XBP-1 (X-box binding protein 1) [11]. We analyzed the sequence of *xbp*-1 gene of *C. elegans* and *A. aegypti* and found out that the 23 bp sequence of the intron that is cleaved out during RNA-splicing is highly conserved, showing 60% identity (Figure 1A). In order to analyze if intoxication with Cry11Aa could activate IRE-1, we analyzed the splicing event of *xbp*-1 mRNA by RT-PCR. Twenty fourth-instar larvae were fed with Cry11Aa for 2 h using a concentration of toxin that kills 50% of the population in 24 h and compared with control larvae without toxin. As a positive control we used tunicamycin that is a natural compound that leads to accumulation of unfolded proteins and functions as an activator of IRE-1 [13]. Figure 1B shows that treatment of *A. aegypti* larvae with Cry11Aa induced the splicing of *xbp-*1 mRNA similar to the treatment with tunicamycin, indicating that Cry11Aa intoxication activates the IRE-1 branch of UPR.

**Figure 2:**
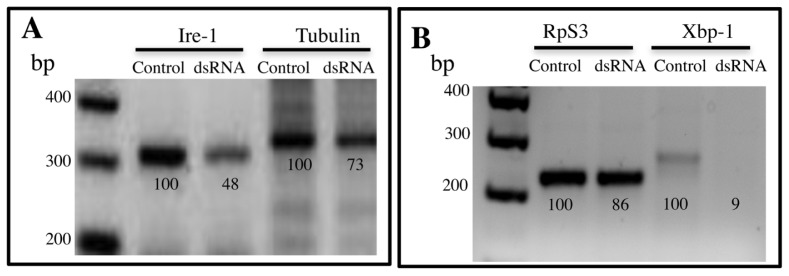

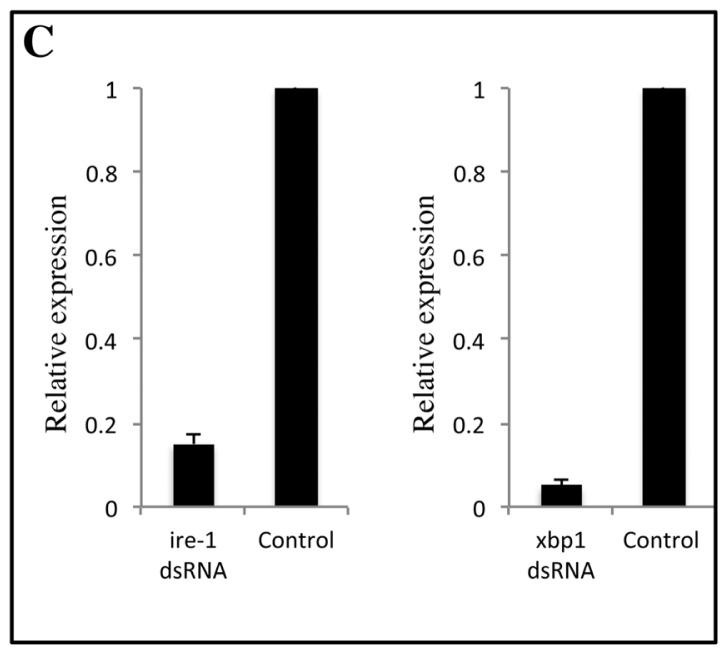
Silencing of IRE-1 branch of UPR pathway by RNAi in *Aedes aegypti* larvae. (**A**) The expression of *ire*-1 gene was analyzed by RT-PCR assays in larvae that were feed with *ire*1-dsRNA and in control larvae; (**B**) The expression of *xbp*-1 gene was analyzed by RT-PCR assays in larvae that were feed with *xbp*1-dsRNA and in control larvae. Numbers under the bands are percentage in relation to the control band, after densitometry analysis. The control bands correspond to non-silenced larvae and were considered as 100%. The expressions of tubulin or rpS3 were used to normalize the results; (**C**) Transcript abundance was determined using qRT-PCR and SYBER green. Bars represent the means and standard errors of three repetitions.

To determine if UPR pathway plays a role in the defense of *A. aegypti* against Cry11Aa toxin *in vivo*, we silenced the expression of IRE-1 and XBP-1 proteins by using RNAi. We cloned two fragments of *ire-*1 and *xbp-*1 genes in pLITMUS and produced specific dsRNA of these genes. The silencing of *ire-1* resulted in a lower transcription levels of *ire-*1 (35% lower expression), as shown in the RT-PCR analysis (Figure 2A). RNA transcript levels were also determined by real-time quantitative PCR (qPCR) as described in Experimental section showing an 82% reduction of *ire*-1 transcripts (Figure 2C). The low expression of *ire-*1 resulted in a hypersensitive phenotype to Cry11Aa intoxication since larvae became more sensitive to Cry11Aa toxin showing an LC_50_ value that was 2.6 times lower than that of control larvae (Table 1). We observed 80% surviving of silenced larvae up to the 4th instar and 95% surviving in the control larvae that were fed only with Effectene-vesicles without dsRNA. Nevertheless, it is important to mention that the *ire-1* silenced larvae grew similar to the control larvae and they looked healthy under control conditions without toxin intoxication.

In order to confirm the participation of UPR pathway in defense mechanism against Cry11Aa intoxication in the mosquito, the effect of silencing of XBP-1 protein in the susceptibility of *A. aegypti* to Cry11Aa toxin was also analyzed. The silencing of the expression of this protein was quite effective since we observed a reduction of 93% in the levels of *xbp-*1-mRNA (Figure 2B). RNA transcript levels determined by qPCR confirmed a reduction of *xbp*-1 transcripts of 95% (Figure 2C). We found that silencing this protein did not affect severely the larvae development since 83% of the silenced larvae survived up to the 4th instar and look healthy without toxin administration. The only difference in this case, was that the larvae grew more slowly than the control and required 3–5 more days to reach the 4th instar. The bioassays performed with the *xbp*-1 silenced larvae showed 3.1 fold higher sensibility to the toxin, confirming the participation of UPR pathway in defense against Cry11Aa intoxication (Table 1).

### 2.2. Role of SREBP Pathway in Response to Cry11Aa in *A. aegypti*

**Figure 3:**
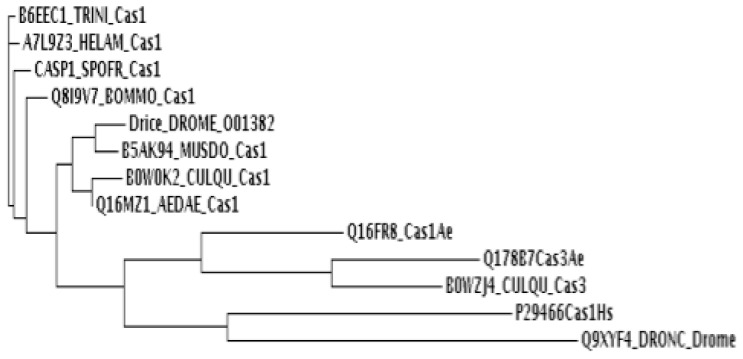
Phylogenetic tree of Cas-1 proteins from different insects and from humans. A phylogenetic tree was constructed using the following sequences: Cas-1 from *Bombyx mori* (Q8I9V7), *Spodoptera frugiperda* (P89116), *Trichoplusia ni* (B6EEC1) *Helicoverpa armigera* (A7L9Z3), *Drosophila melanogaster* (O01382), D. *melanogaster* (Q9XYF4), *Culex quinquefasciatus* (B0W0K2), *Musca domestica* (B5AK94), *Aedes aegypti* (Q16MZ1), *A. aegypti* (Q16FR8), *Homo sapiens* (P29466) and Cas-3 from *A. aegypti* (Q178B6) and *C. quinquefasciatus* (B0WZJ4).

It was reported that in mammals, caspase 1 is involved in activation of SREBP pathway after intoxication with the pore forming toxin aerolysin [7]. Specifically in the case of *D. melanogaster* it was shown that caspase-Drice is directly involved in the activation of SREBP [14]. We performed a sequence alignment and phylogenetic analysis of caspase-1 sequences from insects, including the sequence of caspase-1 from humans. We found out that caspase-1 (Q16MZ1) from *A. aegypti* has 74% identity with caspase-Drice from *D. melanogaster* and 79% identity with Caspase-1 from *Anopheles gambiae* (Figure 3 and Table 2). We also found the orthologous proteins of S2P and SCAP wich showed specially high identity with S2P and SCAP proteins from *Culex quinquefasciatus* (Table 2) and decided to analyze the effect of silencing these proteins in *A. aegypti* regarding to larval sensibility to Cry11Aa toxin.

**Figure 4:**
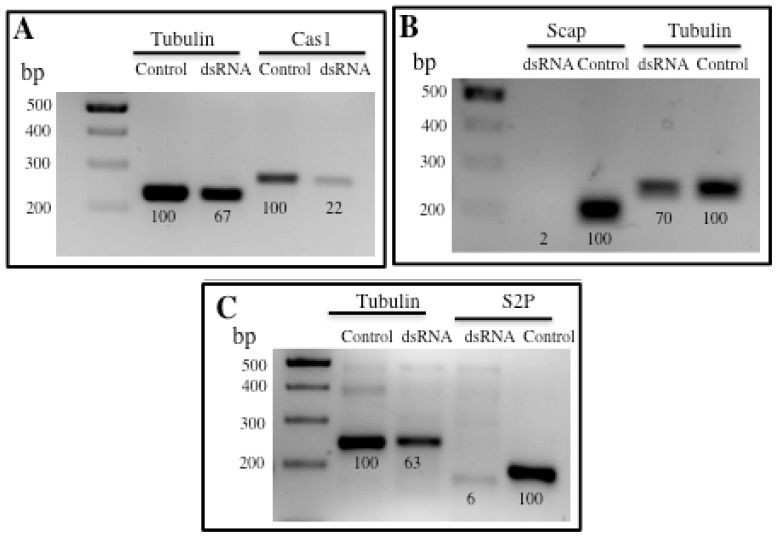
Silencing of SREBP pathway by RNAi in *Aedes aegypti* larvae. (**A**) The expression of *cas*-1 gene was analyzed by RT-PCR assays in larvae that were feed with *cas*1-dsRNA and in control larvae; (**B**) The expression of *scap* gene was analyzed by RT-PCR assays in larvae that were feed with *scap*-dsRNA and in control larvae; (**C**) The expression of *s2p* gene was analyzed by RT-PCR assays in larvae that were feed with *s2p*-dsRNA and in control larvae. Numbers under the bands are percentage in relation to the control band, after densitometry analysis. The control bands correspond to non-silenced larvae and were considered as 100%. Expression of tubulin was used to normalize the results.

The silencing of Caspase-1 in *A. aegypti* resulted in 64% reduction of the expresion of its mRNA (Figure 4A). This silencing did not affect the viability of the larvae since they could survive up to the 4th instar similar to the control larvae. The susceptibility to Cry11Aa toxin increased 1.4 fold, but this change was not significant since confidential limits overlap (Table 1). On the other side, silencing of SCAP and S2P was effective showing 91% and 97% reduction in their corresponding mRNA levels (Figure 4B,C). Silencing of these genes did not affect the susceptibility to Cry11Aa toxin in *A. aegypti* since LC_50_ values observed showed a clear overlap in the confidence interval values, suggesting that differences in LC_50_ values were not significant (Table 1). However, it is interesting to note that the absence of SCAP we observed reduced viability of the larvae to 62% suggesting that SREBP pathway is important in maintaining the homeostasis of the lipid content in the membrane.

## 3. Discussion

The UPR system is a complex response to stress situations in the cell. It has been shown that one of the transducer-branches that activates UPR response, named IRE-1, is specifically activated after treatment *C. elegans* nematodes with Cry5Ba and it was proposed that cells have adapted the UPR pathway to promote cellular defense to the toxin by increasing phospholipid biogenesis [5]. IRE-1 is the most conserved branch of UPR in lower eukaryotes [15]; it is a transmembrane protein that has a dual function as kinase/endonuclease [11]. IRE-1 forms dimers that are activated by auto-phosphorylation and cleaves out the mRNA of the specific transcription factor XBP-1 in two sites, excising an intron of 23 nucleotides [11]. The activated form of XBP-1 has a role in regulating lipid biosynthetic enzymes and ER-associated degradation components [16,17].

We show here that the sequence of the *xbp*1-intron that is cleaved out by IRE-1 is conserved between *C. elegans* and *A. aegypti*. We also show that *xbp*1-intron is cleaved after larvae were intoxicated with Cry11Aa, suggesting that IRE-1 branch of UPR is activated by treatment with Cry11Aa toxin. The role of UPR in defense response against Cry11Aa toxin was demonstrated by silencing the expression of IRE-1 and XBP-1 proteins by RNAi. Previously, we demonstrated that the silencing of another protein (P38 MAPK) could be performed in the mosquito larvae after feeding with the corresponding dsRNA encapsulated in Effectene-vesicles [4]. It is important to mention that the *ire-1* and *xbp-1* silenced larvae grew similar to the control larvae and they seem healthy under control conditions without toxin intoxication. In the case of *C. elegans* it was reported that mutant worms in *ire*-1 gene were smaller than the wild type animals suggesting that this protein is important for the viability of the nematodes [5]. One advantage of RNAi *vs*. gene knockout is that in RNAi the expression of silenced protein is reduced but not completely eliminated, and sometimes this is an advantage to avoid lethal or non-healthy phenotypes. The 4th instar *ire*-1 or in *xbp-*1 silenced larvae were tested in bioassays with Cry11Aa toxin showing that they became more sensitive to Cry11Aa toxin action (Table 1).

In *C. elegans* it was reported that UPR is activated in response to MAPK p38 [5]. A mutation in p38 in *C. elegans* resulted in 100-fold increase in sensitivity to Cry5Ba toxin. In contrast a *C. elegans* mutant in *ire*-1 became 13-fold more sensitive to Cry5B toxin, while the silencing of *xbp*-1 resulted in 5.7 fold increase in sensitivity [5]. The differences in sensitivity to Cry5B between the *ire-*1 and p38 mutants suggest that MAPK p38 activates other defense mechanisms besides UPR. In the case of the mosquito *A. aegypti* the silencing of p38 resulted in 10 fold increase in sensitivity [4] while the silencing of *ire*-1 or *xbp-*1 resulted in 2.6 and 3.1 fold increase in susceptibility to Cry11Aa, respectively, suggesting that both p38 and UPR pathways are participating in the defense mechanisms in the mosquito. It remains to be determined if UPR activation is part of the response that is activated by p38 MAPK pathway in *A. aegypti*.

Insects cannot synthesize cholesterol so they have the requirement to eat incorporated sterols in their diet. However the SREBP pathway is highly conserved in insects where it is involved in lipid metabolism. In *Drosophila melanogaster* a single SREBP gene was reported as well as single orthologous genes for S1P, S2P and SCAP [18,19]. Mutants in these genes can be rescued by supplementing their diet with fatty acids [20]. It was reported that in mammals, caspase 1 is involved in activation of SREBP pathway. In the case of *D. melanogaster*, Caspase-Drice was shown to be involved in the activation of SREBP [14]. We identified caspase-1 (Q16MZ1) from *A. aegypti* to correspond to caspase-Drice from *D. melanogaster*. The silencing of Caspase-1 (Q16MZ1), S2P and SCAP in *A. aegypti* did not affect viability of the larvae neither their susceptibility to Cry11Aa toxin suggesting that SREBP pathway does not play a major role in defense mechanism against Cry11Aa pore forming toxin. These data are in contrast to the previous report that clearly showed that in mammalian cells SREBP pathway has an important role in a defense mechanism against the action of aerolysin pore forming toxin [7]. However, it is important to mention that it was reported that dScap and dS2P, which are essential components of the SREBP activation machinery in mammalian cells, are dispensable in *D. melanogaster* owing to different compensatory mechanisms [21]. This could also be the case of Scap and S2P in *A. aegypti* since larvae that were silenced in these genes were viable such a *Drosophila* flies that lack dSCAP and dS2P which are viable and are able to cleave dSREBP. Due to the fact that SREBP system in mosquitoes is more related to *D. melanogaster* than to mammalians, it is possible that compensatory mechanisms may also be present in *A. aegypti*. Thus, the silencing of these individual genes would not prevent the complete cleavage of SREBP and it would not be possible to correlate the silencing of expression of these genes with the complete blocking of the SREBP pathway. Matthews *et al.* (2010) [21] proposed two alternative means of activating SREBP in flies: the first, requiring cleavage of SREBP by the caspase-Drice, would explain the survival of flies lacking dS2P, and the second, suggest that SREBP cleavage could be done by dS1P and dS2P in a subset of tissues in the absence of dSCAP. Due to these arguments, we cannot conclude that SREBP pathway does not play a role in the defense mechanism against pore forming Cry11Aa toxin in *A. aegypti* and more experiments should be done in the future.

## 4. Experimental Section

### 4.1. Production of Cry11Aa Crystal Inclusions

For the production of Cry11Aa crystals, acrystalliferous Bti strain Q2-81 containing plasmid pGC6 [22] were cultured for 3 days at 29 °C in Petri-dishes containing solid nutrient broth sporulation medium supplemented with erythromycin (25 μg/mL). Crystal inclusions were observed under phase contrast microscopy and the spores and inclusion bodies were harvested and washed three times with 0.3 M NaCl, 0.01 M EDTA, pH 8.0. Finally, crystal/spore samples were suspended in water supplemented with 1 mM PMSF.

### 4.2. RNA Interference (RNAi) Assays

The midgut tissue of 30 larvae in the early 4th instar were dissected and stored at −80 °C in RNA*later* (QIAGEN, Hilden, Germany). Total RNA was purified using RNeasy Kit (QIAGEN, Hilden, Germany). The RNA was quantitated in a NanoDrop2000 Thermo Scientific spectrophotometer, (Frankfurt, Germany). First strand cDNA was synthetized using 1 μg of total RNA and oligo dT as described in the Synthesis SuperScript II Reverse Transcriptase Kit (Invitrogen, Grand Island, NY, USA). Selected genes were: Ire-1 (Uniprot accession: XM_001655187.1); Xbp-1 (Uniprot: Q179N5, accession: XM_001651044.1); Cas-1 (Uniprot: Q16MZ1, accession: XM_001655826.1); Scap (Uniprot: Q17N28, accession: XM_001651241.1); S2P (Uniprot: Q16J11, accession: XM_001663618.1). Fragments from each selected gene were amplified from *A. aegypti* cDNA using the sense and antisense primers described in Table 3, which contain *EcoR*I and *Hind*III restriction sites at their 5′ ends. The sequence of these PCR products was verified by DNA sequence in the facilities of Institute of Biotechnology, UNAM. The PCR products were digested with *EcoR*I and *Hind*III restriction enzymes and cloned into pLitmus28i vector (HiScribeTM, New England Biolabs, Beverly, MA, USA) containing two T7 promoters flanking the multi-cloning site. Ligation mixtures were transformed into TOP10 *Escherichia coli* cells and selected in Luria broth medium supplemented with 100 μg/mL ampicillin. The pLitmus28i plasmids containing each of the selected genes were purified using QIAprep Miniprep Kit (QIAGEN). Each of the selected genes was then amplified by PCR using T7 oligonucleotides and the PCR product was purified with MiniElute columns (QIAGEN). *In vitro* transcription of both DNA strands of the insert was performed with T7 RNA polymerase using the HiScribe RNAi Transcription Kit (New England Biolabs) as reported by the manufacturer, yielding dsRNA.

For RNA silencing, 300 neonate larvae were fed during 16 h with 2.5 μg of dsRNA per larvae previously encapsulated with Effectene transfection reagent (QIAGEN) in a total volume of 15 mL. After 16 h of feeding the final volume was adjusted to 1 L with dechlorinated water and fed with regular diet until they reached the 4th instar when bioassays were performed or guts were dissected for RT-PCR analyses.

### 4.3. RT-PCR and Quantitative Real Time PCR

Total RNA was isolated and cDNA was synthetized as described above from control larvae or from larvae that were feed with dsRNA of each gene. Specific oligonucleotides (Table 3) were used to amplify by RT-PCR each *A. aegypti* gene. Amplification of tubulin alpha chain (Uniprot: Q1HR53, accession: XM_001652094.1) or rpS3 (Uniprot: J9HFW1, accession: XM_001658973) were used as controls. Amplified products were observed in 2% agarose gel and intensity of the bands was determined by densitometry using ImageJ program. For analysis of *xbp-1* splicing, electrophoresis was performed in 4% agarose gels at 110 V. Tunicamicyn was used at 10 μg/mL during 2 h. Quantitative real-time PCR was performed on each template using primers listed in Table 3, on a Light Cycler 480 Instrument (Roche, Basel, Switzerland) using Sybr Green I Detection System (Fermentas, Life Sciences, Waltham, MA, USA). Relative-fold calculations were made with triplicates for each treatment-group analyzing *rps3* (ribosomal protein S3) gene to normalize gene expression.

### 4.4. Insect Bioassays

To determine the mean lethal concentration (LC_50_) of Cry11Aa, ten early 4th instar *A. aegypti* larvae reared at 28 °C, 87% humidity and 12:12 light: dark period were placed in 100 mL dechlorinated water. Cry11Aa spore-crystal suspensions (from 50 to 3000 ng/cm^2^) were directly added to the 100 mL H_2_O. Mortality was recorded after 24 h and lethal concentration (LC_50_) values were estimated by Probit (Polo-PC LeOra Software, Petaluma, CA, USA) using data of three repetitions. Protein concentration was determined by the Bradford assay.

### 4.5. Phylogenetic Analysis

The virtual translations of *cas-1* gene sequences from different insects and also the *cas-1* from humans were aligned using Muscle 3.7 alignment [23]. A maximum likelihood tree was constructed and drawn using PhyML version 3.0 [24] with a bootstrap of 500 replicates.

## 5. Conclusions

The data presented here show that in *A. aegypti*, UPR is involved in the defense mechanism against Cry11A toxin. The identification of this defense mechanism to Cry toxins is likely to provide cellular targets to improve the insecticidal activity of these important biotechnological and useful toxins.

## Figures and Tables

**Table 1:** Percentage of larval survival up to 4th instar after silencing and toxicity of spore-crystal suspensions of Cry11Aa against *Aedes aegypti* larvae.

Silenced protein	Larval survival % b	LC_50_ value (95% fiducial limits)	Fold increase in susceptibility
Control	95	555 (408–725)	-
IRE-1	80	214 (137–306)	2.6
XBP-1	83	176.4 (127.1–222.6)	3.1
CASP1(Q16MZ1)	98	392.7 (230.6–542.4)	NS a
SCAP	6	683 (475–1160)	NS
S2P	87	355 (137–533)	NS

a NS, non-significant changes with the control larvae as estimated by Probit analysis of confidence intervals since the fiducial limits are overlapping;

b Differences in percentage of survival were 1%–5%.

**Table 2:** Percentage of identity with similar proteins described in other insects.

Protein (Accession number)	Percentage of identity (Accession number)		
	
*Aedes aegypti*	*Culex quinquefasciatus*	*Anopheles gambiae*	*Drosophila melanogaster*
Ire-1 (XM_001655187.1)	77% (XP_001843113)	61% (XP_562694)	55% (NP_001097839)
Xbp-1 (XM_001651044.1)	66% (XP-001847153)	61% (XP-310116)	46% (NP-524722)
Cas-1 (XM_001655826.1)	94% (XP-001842236)	79% (XP-316795)	74% (CAA72937)
Scap (XM_001651241.1)	89% (XP_001863686)	74% (XP_309314)	54% (AAM20923)
S2P (XM_001663618.1)	84% (XP_001663668)	68% (XP_320696)	46% (NP_610705)

**Table 3:** Sequence of oligonucleotides.

Gene	Oligonucleotide sequence	PCR product size (bp)
S2P	5′-CCG GAA TTC AAC ATT CGG AAG GTG ATT GA-3′ 5′-CCC AAG CTT GGT GGC CAA TGT AGA TAA CG-3′	172
SCAP	5′-CCG GAA TTC GTG GGA TAA GTC GTT CGA TG-3′ 5′-CCC AAG CTT TCA TGA AGC CTC TTT GGA AG-3′	192
CASP1Q16	5′-CCG GAA TTC TAT CTG TAT GCA AAG GA-3′ 5′-CCC AAG CTT ATG AGT AGA ATC CCG GAA TG-3′	268
XBP-1	5′-CCG GAA TTC TCA ACG ATC TTC AGC AGC AC-3′ 5′-CCC AAG CTT TGT AGA GCA GGC AGA GAG CA-3′	264
IRE-1	5′-CCG GAA TTC TGC TGT TGC AAA AGA TGA GG-3′ 5′-CCC AAG CTT CTC AGG ATT CCG GTA CGT GT- 3′	220
TUBULIN	5′-CTA CGG CAA GAA GTC CAA GC-3′ 5′-GAA GCG GTG ATC GAA GAG AC-3′	243
RPS3	5′-TTC TCG GCG TAC AGC TCG ACG-3′ 5′-GGC ATG TTC CGT GCT GAA TTG AAC G-3′	239
