# Addendum: Bedoya-Pérez, L.P. et al. Role of UPR Pathway in Defense Response of *Aedes aegypti* against Cry11Aa Toxin from *Bacillus thuringiensis*. *Int. J. Mol. Sci.* 2013, *14*, 8467–8478

**DOI:** 10.3390/ijms17122021

**Published:** 2016-12-02

**Authors:** Leidy P. Bedoya-Pérez, Angeles Cancino-Rodezno, Biviana Flores-Escobar, Mario Soberón, Alejandra Bravo

**Affiliations:** Instituto de Biotecnología, Universidad Nacional Autónoma de México. AP 510-3, Cuernavaca 62250, Morelos, Mexico; lbedoya@ibt.unam.mx (L.P.B.-P.); angelescancino@gmail.com (A.C.-R.); biviana@ibt.unam.mx (B.F.-E.); mario@ibt.unam.mx (M.S.)

The authors would like to indicate that Dr. Angeles Cancino-Rodezno and Leidy P. Bedoya-Pérez participated equally in their paper published in the *International Journal of Molecular Sciences* [1]. For this reason both should be considered as co-first authors. The authors apologize for any inconvenience this change may cause.

The changes do not affect the scientific results. The manuscript will be updated and the original will remain online on the article webpage.
